# Etiology, treatment outcome and prognostic factors among patients with secondary peritonitis at Bugando Medical Centre, Mwanza, Tanzania

**DOI:** 10.1186/s13017-015-0042-5

**Published:** 2015-10-06

**Authors:** Amri Mabewa, Jeremiah Seni, Phillipo L. Chalya, Stephen E. Mshana, Japhet M. Gilyoma

**Affiliations:** Department of Surgery, Catholic University of Health and Allied Sciences, P.O. Box 1464, Mwanza, Tanzania; Department of Surgery, Bugando Medical Centre, P.O. Box 1370, Mwanza, Tanzania; Department of Microbiology and Immunology, Catholic University of Health and Allied Sciences, P.O. Box 1464, Mwanza, Tanzania

**Keywords:** Etiology, Treatment outcome, Prognostic factors, Secondary peritonitis, Tanzania

## Abstract

**Introduction:**

Secondary peritonitis due to perforation of the gastrointestinal tract is one of the most common surgical emergencies all over the world and is associated with significantly morbidity and mortality. Previous studies conducted at Bugando Medical Centre (BMC) were retrospective and each was focused on single etiology; therefore there was an obvious need to evaluate the etiologies, treatment outcome and their prognostic factors altogether.

**Methods:**

This was a descriptive cross-sectional study involving patients with secondary peritonitis admitted at BMC from May 2014 to April 2015. Sociodemographic and clinical characteristics among consented patients were collected using questionnaires. Peritoneal aspirate, biopsy and blood were collected perioperatively and processed using standard operating procedures. Analysis was done using STATA version 11 software.

**Results:**

The study enrolled 97 patients with the female to male ratio of 1:1.8 and approximately 41.2 % (40/97) were in their third and fourth decades of life. Only 3 (3.09 %) patients arrived to the hospital within 24 hours of onset of illness, 26 (26.80 %) patients presented with shock and HIV seropositivity among all patients was 13.40 % (13/97). The common etiologies of secondary peritonitis were perforated appendicitis 23 (23.71 %), peptic ulcer disease 18 (18.56 %), ischemia 18 (18.56 %) and typhoidal perforation 15 (15.46 %). Of the 97 patients, 35 (36.08 %) had complications and 15 (15.46 %) died. Presence of premorbid illness and post-operative complication were found to be associated with death (*p* values = 0.004 and <0.001 respectively).

**Conclusions:**

The most common etiologies of secondary peritonitis at BMC are perforated appendicitis, peptic ulcer disease, ischemia and typhoidal perforation. Premorbid illness and postoperative complications in this setting are associated with death and as the matter of fact proper screening on admission should be done to identify patients with premorbid illness and confer prompt management.

## Introduction

Peritonitis has a long historical background and is conventionally divided into three broad groups namely; primary, secondary and tertiary basing on the source and nature of the microbial contamination [1]. Secondary peritonitis which is due to perforation of the gastrointestinal tract is one of the most common surgical emergencies all over the world and it is associated with significantly morbidity and mortality [1–4]. Due to the loss of epithelial integrity, bacterial pathogens can traverse into the peritoneal cavity leading to a cascade of inflammatory response, sepsis, multisystem organ failure and death if not treated in a timely manner [1]. Aggressive resuscitation and early surgical intervention is therefore required in order to deliver optimal care for the patients and improve their treatment outcome [2–5].

The spectrum of gastrointestinal perforation have a wide geographical variations; with preponderance of lower gastrointestinal perforations western countries as opposed to upper gastrointestinal perforations in developing countries [3, 6–8].

At Bugando Medical Centre (BMC), primary bacterial peritonitis was recently reported to have a prevalence of 11.6 % among patients with portal hypertension and ascites [9]. Moreover, secondary peritonitis is the commonest indication for admission in the surgical wards resulting into increased workload, increased duration of hospital stays, and complications such as enterocutaneous fistula, surgical site infections and sepsis. Furthermore, the death rates attributable to secondary peritonitis in this settings has been shown to be 10.7 % and 23.1 % among patients with peptic ulcer and typhoid perforations respectively [10–12]. Despite advances in surgical techniques, antimicrobial therapy, and intensive care support; the management of peritonitis continues to be highly demanding, difficult, and complex [3, 13]. Early prognostic evaluation of peritonitis is desirable to provide objective classification of the severity of the disease and select high-risk patients for more aggressive therapeutic procedures [14, 15]; but this has been affected by late presentation to the health facilities by majority of patients, a situation which further complicates effective management [16].

Previous studies from BMC were retrospective and each focused on single etiology thus, lacking a holistic approach in addressing the implicated etiologies [10–12]; therefore the present study aimed at exploring the wide range of etiologies causing secondary peritonitis so as to identify factors responsible for the poor outcome and specifically address them in the context of reducing poor outcomes among patients with secondary peritonitis.

## Methods

### Study design, site and sampling procedures

A descriptive cross-sectional study involving patients with secondary peritonitis admitted at BMC from May 2014 to April 2015 inclusive was conducted. BMC is one of the four largest referral hospitals in the country, located in the northwestern part of Tanzania. It has a bed capacity of 1000 and serves a catchment population of approximately 13 million people. It is also a consultant and teaching hospital for the Catholic University of Health and Allied Sciences-Bugando (CUHAS). A minimum sample size of 93 was estimated basing on the Yamane Taro (1967) using previous local data which revealed 243 patients who were operated with secondary peritonitis in 2013 as well as accepted error of 8 %. Patients who met the inclusion criteria were offered explanations about the study and requested to provide a written informed consent before being enrolled into the study.

Preoperatively, all the patients recruited into the study were resuscitated with intravenous fluids to correct fluid and electrolyte deficits; nasogastric suction; urethral catheterization and broad-spectrum antibiotic coverage as per BMC protocol. Relevant preoperative investigations included hemoglobin estimation, blood grouping and cross-matching. In addition, provider initiated testing for HIV infection was offered to all patients according to National AIDS Control Program [17, 18]. For patients found to be HIV seropositive, CD4+ count using FACSCALIBUR (BD Biosciences, USA) was done. Radiological investigations included abdominal x-rays (erect and supine) as well as abdominal ultrasound were performed in all patients.

After resuscitation, all patients had to undergo pre-operative anesthetic assessment using the American Society of Anesthetists (ASA) classification [19]. Patients who were clinically and hemodynamically stable for surgery were subjected to exploratory laparotomy under general anesthesia by the residents using the standard techniques.

The diagnosis of secondary peritonitis was established clinically based on guarding, rigidity, and tenderness on palpation of the abdomen, supported radiologically by detection of free air under the diaphragm and/or free intra peritoneal fluid on abdominal ultrasound and confirmed by intraoperative clinical and investigational findings.

Intraoperatively, purulent materials were aspirated from peritoneal cavity for Ziehl-Neelsen stain for Acid fast bacilli (AFB), tissue biopsy and regional lymph nodes were taken for histopathological analysis [20]. The primary outcome of the study was survival i.e. whether a patient died or was alive at the time of discharge.

### Data management

A structured questionnaires were used to collect socio-demographic and clinical data preoperatively, intraoperatively and post-operatively. Data were entered into Microsoft excel and then exported to the STATA version 11.0 software for analysis according to the objectives of the study. The results are presented into percentages/proportions for categorical variables whereas continuous variables are described as mean (± standard deviation) or median (interquantile range) depending on the distribution of data. The difference in distribution of a predictor variable was considered significant if *p*-value is less than 0.05.

### Ethical considerations

Ethical clearance was obtained from the joint BMC/CUHAS ethical committee. Written informed consent was obtained from every participant prior to be involved in the study. Anonymity of patients was ensured by use of codes. Laboratory results were timely reported to the attending doctors for specific management.

## Results

A total of 112 patients were diagnosed to have secondary peritonitis and were admitted during the study period; of these 97 fulfilled the inclusion criteria and were enrolled whereas15 patients were excluded (samples were not taken in 10 and 5 did not fulfill inclusion criteria).

The female to male ratio of the study participants was 1:1.8. The median age (IQR) of the study was 32 (21–47) years, the youngest was 5 years old and the oldest was 86 years old. Moreover, approximately 41.2 % (40/97) were in their third and fourth decades of life, 51 (52.58 %) were from urban, and majority had primary school education (59.79 %) and were unemployed (62.89 %).

### Clinical presentations

Majority of patients (96.91 %, 94/97) presented to hospital more than 24 hours since the illness started. Twenty seven (27.84 %) patients presented with co morbid illness, with HIV infection being the predominant co morbidity in about 46.43 % (13/28) of patients. Abdominal pain was found to be the most common presenting symptom in about 95 (97.6 %) patients and 93 (95.88 %) were diagnosed with generalized peritonitis. About 26 (26.80 %) patients presented with shock with systolic blood pressure bellow 90 mmHg and heart rate was above 100 beats per minute and 56 (57 .73 %) patients were febrile (Table 1).

**Table 1:** Clinical presentation of the patients with secondary peritonitis at BMC

Characteristic			Number (%)
Symptoms	Vomiting	Yes	30 (30.93)
		No	67 (69.07)
	Abdominal pain	Yes	95 (97.94)
		No	2 (2.06)
	Fever	Yes	65 (67.01)
		No	32 (32.99)
	Diarrhea	Yes	3 (3.09)
		No	94 (96.91)
	Constipation	Yes	17 (17.53)
		No	80 (82.47)
	Dehydration	Yes	2 (2.06)
		No	95 (97.94)
Type peritonitis	Generalized		93 (95.88)
	Localized		4 (4.12)
Signs	Systolic BP	≤90 mmHg	26 (26.80)
		>90 mmHg	71 (73.20)
	Heart rate	Below 100 bpm	30 (30.93)
		Above 100 bpm	67 (69.07)
	Respiratory rate	Below 30 brpm	87 (89.69)
		Above 30 brpm	10 (10.31)
	Temperature	Below 36 °C	2 (2.06)
		36-38 °C	39 (40.21)
		Above 38 °C	56 (57.73)
Comorbid illness^a^	Yes		27 (27.84)
	No		70 (72.16)

^a^HIV (12); Puerperal sepsis (4); Severe anemia (4); Hypertension (4); Tumor (1); Heart failure (1); Renal failure (1) and HIV & Hypertension (1)

### Laboratory and radiological investigations

The median hemoglobin (IQR) of the 97 patients was 9.2 (7.3–11) gm/dl; ranging from 1.2 gm/dl to 17 gm/dl. Widal test was done in 71 patients and titers were high in 14 patients and were within normal range in 57 patients. Thirteen patients (13.40 %) were found to be HIV seropositive; with the median CD4 count of 250 (150–330) and lowest level was 95 and highest was 480.

Abdominal x ray supine and erect were normal in 51 (52.58 %) patients and abnormal in 46 (47.42 %). Pneumoperitoneum was found in 31 (31.96 %) and air fluid level in 35 (36.08 %) patients (Table 2).

**Table 2:** The investigation findings of patients with secondary peritonitis at BMC

Investigations		Number (%) or (IQR)
Hemoglobin		9.2 (7.3-11) g/dl
Widal test	Suggestive	14 (19.72)
	Not suggestive	57 (80.28)
HIV	Reactive	13 (13.40)
	Non reactive	84 (86.6)
CD_4_^a^		250 (150–330)
X ray	Normal	51 (52.58)
	Abnormal	46 (47.42)
Ultrasound	Fluid seen	32 (32.99)
	No fluid	3 (3.09)
	Not done	62 (63.92)

^a^Only HIV positive patients were included

### Operative data

Majority of the patients were in ASA 2 and ASA 3 in about 58 (59.79 %) and 24 (24.74 %) respectively. The median waiting time (IQR) was 3 (2–6) hours. The common etiologies of secondary peritonitis were perforated appendicitis 23 (23.71 %), peptic ulcer disease 18 (18.56 %), ischemia 18 (18.56 %) and typhoidal perforation 15 (15.46 %). The median size of perforation (IQR) was 1 (0.8-1) cm. The minimum size perforation was 0.4 cm and maximum size was 3 cm.

The most common surgical interventions done were bowel resection and anastomosis in 27 (27.84 %), appendectomy in 21 (21.65 %), Grahams omental patch in 18 (18.56 %) patients and perforation were repaired in 16 (16.49 %). The median time for operation (IQR) was 2 (1.5-2) hours, with the shortest time being 45 minutes and longest time was 4 hours (Table 3).

**Table 3:** The operative findings of patients with secondary peritonitis

Characteristic	Number (%) or median (IQR)	
Timing of operation	Less than 24	3 (3.09)
	More than 24	94 (96.96)
Waiting time	3(2–6) hours	
ASA	1	8 (8.25)
	2	58 (59.79)
	3	24 (24.74)
	4	6 (6.19)
	5	1 (1.03)
Site of perforation	Gastric	18 (18.56)
	Small intestine	28 (28.87)
	Appendix	23 (23.71)
	Large intestine	18 (18.56)
	Others^a^	10 (10.31)
Etiology of perforation	PUD	18 (18.56)
	Typhoid	15 (15.46)
	Appendicitis	23 (23.71)
	Trauma	11 (11.34)
	Neoplastic	2 (2.06)
	Ischemia	18 (18.56)
	Others^a^	10 (10.31)
Type of surgery	Repair of perforation	16 (16.49)
	Resection and anastomosis	27 (27.84)
	Grahams’ omental patch	18 (18.54)
	Appendectomy	21 (21.65)
	Others^b^	15 (15.46 %)
Duration of operation	2(1.5-2) hours	

^a^Ruptured abscess (9) and Perforated gall bladder (1)

^b^Cholecystectomy (1); Diverting stoma (1) and Lavage (13)

### The outcome of treatment

Of the 97 patients, 35 (36.08 %) had complications, of which 18 (51.43 %) had surgical site infections, 11 (31.43 %) wound dehiscence and 6 (17.14 %) developed septicemia. The median length of hospital stay (IQR) was 7 (5–11) days; the longest hospital stay was 60 days and shortest 1 day. Mortality rate was 15 (15.46 %) (Table 4).

**Table 4:** Outcome of treatment for the patients with secondary peritonitis at BMC

Parameter		Number (%) or median (IQR)
Complications	Yes	35 (36.08)
	No	62 (63.92)
Type of complication	Surgical site infection	18 (18.55)
	Wound dehiscence	11 (11.34)
	Septicemia	6 (6.19)
Survival	Yes	82 (84.54)
	No	15 (15.46)
Length of hospital stay (days)		7 (5–11)

### The prognostic factors for mortality

There was no significant association between socio-demographic characteristics of patients with the outcome. Presence of premorbid illness and post-operative complications were found to be significantly associated with mortality (*p* value of 0.004 and <0.001 respectively) (Tables 5 and 6).

**Table 5:** Association of socio-demographic characteristics with outcome among patients with secondary peritonitis at BMC

Predictor variable		Outcome (N = 97)		Chi^2^	p-value
		Survived (82)	Died (15)		
		n (%) or median (IQR)	n (%) or median (IQR)		
Median age (years)		32 (21–45)	34 (31–51)	-	0.4244
Sex	Female	28 (34.15)	7 (46.67)	0.8619	0.353
	Male	54 (65.85)	8 (53.33)		
Residence	Urban	45 (54.88)	6 (40.00)	1.1257	0.289
	Rural	37 (45.12)	9 (60.00)		
Education	Informal	12 (14.63)	2 (13.33)	-	0.444
	Primary	47 (57.32)	11 (73.33)		
	Secondary	23 (28.05)	2 (13.33)		
Occupation	Employed	19 (2317)	1 (6.67)	2.1153	0.347
	Unemployed	50 (60.98)	11 (73.33)		
	Under 18	13 (15.88)	3 (20.00)

**Table 6:** Association of clinical characteristics with outcome among patients with secondary peritonitis at BMC

Predictor variable		Outcome (N = 97)		Chi^2^	p value
		Survived (82)	Died (15)		
		n (%) or median (IQR)	n (%) or median (IQR)		
Duration of illness	≤24 hours	3 (3.66)	0 (0.00)	-	0.601
	>24 hours	79 (96.34)	15 (100.00)		
Temperature	≤36 °C	2 (2.44)	0 (0.00)	-	0.233
	36-38 °C	30 (36.59)	9 (60.00)		
	>38 °C	50 (60.98)	6 (40.00)		
Comorbid illness	Yes	19 (23.17)	9 (60.00)	8.3764	0.004
	No	63 (76.83)	6 (40.00)		
HIV	Reactive	9 (10.98)	4 (26.67)	2.6900	0.101
	Non reactive	73 (89.02)	11 (73.33)		
CD4^a^		315 (203–330)	172.5 (117.5-342.5)	-	0.4398
Hemoglobin ± SD^b^		9.6 ± 2.7 g/dl	8.6 ± 2.4 gm/dl	-	0.1866
X ray	Normal	44 (54.66)	7 (46.67)	0.2486	0.618
	Abnormal	38 (46.34)	8 (53.30)		
Etiology of perforation	PUD	15 (18.3)	3 (20.0)	-	0.491
	Typhoid	11 (13.4)	4 (26.7)		
	Appendicitis	20 (24.4)	3 (20.0)		
	Trauma	9 (11.0)	2 (13.3)		
	Neoplastic	1 (1.2)	1 (6.7)		
	Ischemia	17 (20.7)	1 (6.7)		
	Others	9 (11.0)	1 (6.7)		
Mean waiting time ± SD^b^ (hours)		7.29 ± 1.09	3.667 ± 1.68	-	0.8043
Complication	Yes	23 (28.02)	12 (80.00)	-	<0.001
	No	59 (71.95)	3 (20.00)		

^a^CD4+ count was only done to patients with HIV infection

^b^Standard deviation

## Discussion

### The etiology of secondary peritonitis at BMC

The predominance of perforated appendicitis, peptic ulcer disease, ischemia and typhoidal perforation as the common etiologies of secondary peritonitis in this study are similar to the study done at St. Francis Hospital Nsambya Kampala-Uganda [21] and other two previous studies in the same hospital [10, 11]. Ruptured appendix and ischemia leading to gangrenous sigmoid volvulus may be due to poor health seeking behavior, as shown in this study whereby majority of patients (96 %) came to hospital more than 24 hours after the onset of illness or mismanagement in the lower health facilities. The reason for ruptured peptic ulcer and typhoidal perforations may be due to poor sanitary condition in developing countries, exposing the patients to Salmonella infection and *Helicobacter pylori* infection in the community, which in turn may result into perforations.

### The treatment outcome of secondary peritonitis

Similar to another previous study [22], about 36.08 % of patients with secondary peritonitis in this study developed complications namely superficial surgical site infection, wound dehiscence and septicemia. Mortality rate was found to be 15.46 % and median length of hospital stay was 7 days. These findings are comparable to other studies [8, 22–24] but lower than what were reported in the same hospital four years ago by Chalya et al. [11] which showed complication rate to be 39.4 %, superficial surgical site infection 55.5 %, mortality 23.1 % and median length of hospital stay of 28 days in patients with typhoidal perforation [11]. The difference could be related to the recently renovated modern accident & emergency department and ICU which are well equipped to provide emergence services. Moreover, in the previous studies initial assessments of patients were done by intern doctors as opposed to now where registrars who are experienced on accident & emergency services initially attend the patients. Surgical site infection found in this study (18.55 %) is similar to what is expected in contaminated wounds as reported previously (21 %); but lower than 26 % reported before in the same hospital by Mawala et al. [1, 25]. This could be due to the fact when the previous study was conducted, the department of surgery consisted of general surgery, urology, orthopedic & traumatology as well as otorhinolaryngology which are now separate departments, and thus more care can be offered timely as a result of this decentralization.

### The prognostic factors for mortality among patients with secondary peritonitis

In this study, only premorbid illness and post-operative complications were statistically found to predict deaths. This is possible because less priority can be given to other premorbid illness in case of obvious surgical condition of the patient and thus, these premorbid illnesses may be overlooked resulting into poor outcome [19, 25–27]. As a result, the present study emphasizes on carefully screening of potential premorbid illnesses and also alert care providers to ensure that they make all efforts to prevent postoperative complications. The predominance of HIV among premorbid illnesses in this and previous study in the same hospital [28] worthy to be further explored using a long term prospective study with large sample size so as to specifically delineate the contribution of HIV in the outcome of patients with secondary peritonitis. But from this study, it is sufficing to reiterate that approximately 30.8 % of patients with HIV infections died and their median CD4+ count were relatively low (172.5 cells/mm^3^) compared to HIV infected patients who survived (315 cells/mm^3^). Development of complications postoperatively was strongly associated with death in the present study, similarly another study revealed that development of complication like sepsis can escalate mortality [22]. Sociodemographic factors such as age, gender, clinical symptoms such as vomiting and sign like fever in this study and another related study were not found to predict deaths [23].

### The limitation of the study

The present study diagnosed peptic ulcer disease basing of clinical (site of perforation) and histology. Stool antigen test for *Helicobacter pylori* could have been of interest to ascertain the role of this point-of-care test and this is recommended in future studies.

## Conclusions

The most common etiologies of secondary peritonitis at BMC are perforated appendicitis, peptic ulcer disease, ischemia and typhoid. Premorbid illnesses and postoperative complications in this setting are associated with death and as the matter of fact proper screening on admission should be done to identify patients with premorbid illness and confer prompt management to prevent postoperative complications and subsequent death. Moreover, a study to explore the long-term outcomes of patients with secondary peritonitis is recommended.

## References

[1] Levison ME, Bush LM (2005). Intra-abdominal Infection. In Mandell, Bennett, & Dolin's Principles and Practice of Infectious Diseases. Peritonitis and Intraperitoneal Abscesses.

[2] Simmen H, Heinzelmann M, Largiader F (1996). Peritonitis classification and causes. Dig Surg.

[3] Malangoni MA, Inui T (2006). Peritonitis - the Western experience. World J Emerg Surg.

[4] Agarwall N, Saha N, Srivastava A, Chumber S, Dhar A, Garg S (2007). Peritonitis 10 years experience in a simple surgical unit. Trop Gastroenterol.

[5] Schein M (2002). Surgical management of intra-abdominal infection: is there any evidence?. Langenbecks Arch Surg.

[6] Agarwal N, Saha S, Srivastava A, Chumber S, Dhar A, Garg S (2007). Peritonitis: 10 years' experience in a single surgical unit. Trop Gastroenterol.

[7] Afridi SP, Malik F, Ur-Rahman S, Shamim S, Samo KA (2008). Spectrum of perforation peritonitis in Pakistan: 300 cases Eastern experience. World J Emerg Surg.

[8] Jhobta RS, Attri AK, Kaushik R, Sharma R, Jhobta A (2006). Spectrum of perforation peritonitis in India--review of 504 consecutive cases. World J Emerg Surg.

[9] Mlwati A (2013). Spontaneous bacterial peritonitis among patients with portal hypertension and ascitis attending Bugando Medical Centre.

[10] Chalya PL, Mabula JB, Koy M, McHembe MD, Jaka HM, Kabangila R (2011). Clinical profile and outcome of surgical treatment of perforated peptic ulcers in Northwestern Tanzania: A tertiary hospital experience. World J Emerg Surg.

[11] Chalya PL, Mabula JB, Koy M, Kataraihya JB, Jaka H, Mshana SE (2012). Typhoid intestinal perforations at a University teaching hospital in Northwestern Tanzania: A surgical experience of 104 cases in a resource-limited setting. World J Emerg Surg.

[12] Mabula JB, Chalya PL, McHembe MD, Kihunrwa A, Massinde A, Chandika AB (2012). Bowel perforation secondary to illegally induced abortion: a tertiary hospital experience in Tanzania. World J Emerg Surg.

[13] Marshall JC, Innes M (2003). Intensive care unit management of intra-abdominal infection. Crit Care Med.

[14] Ranju S, Nishant K, Abhijit B, Homay V (2011). Preoperative predictors of mortality in adult patients with perforation peritonitis. Indian Journal of Critical care Medicine.

[15] Billing A, Frohlich D, Schildberg FW (1994). Prediction of outcome using the Mannheim peritonitis index in 2003 patients. Peritonitis Study Group. Br J Surg.

[16] Ersumo T, WM Y, Kotisso B (2005). Perforated peptic ulcer in Tikur Anbessa Hospital: a review of 74 cases. Ethiop Med J.

[17] NACP: National Guidelines for the Management of HIV and AIDS. Ministry of Health of and Social Welfare. The United Republic of Tanzania. In. Fourth edn. Dar-Es-Salaam; 2012

[18] Lyamuya EF, Aboud S, Urassa WK, Sufi J, Mbwana J, Ndugulile F (2009). Evaluation of simple rapid HIV assays and development of national rapid HIV test algorithms in Dar es Salaam, Tanzania. BMC Infect Dis.

[19] Wolters U, Wolf T, Stutzer H, Schroder T (1996). ASA classification and perioperative variables as predictors of postoperative outcome. Br J Anaesth.

[20] Koneman E, Allen S, Janda W, Schreckenberger PP, Lippincott PA (1997). Color Atlas and Textbook of Diagnostic Microbiology.

[21] Ojuka A, Ekwaro L, Kakande I (2015). Causes and Patterns of Peritonitis at St. Francis Hospital Nsambya, Kampala-Uganda. East and Central African Journal of Surgery.

[22] Fukuda N, Wada J, Niki M, Sugiyama Y, Mushiake H (2012). Factors predicting mortality in emergency abdominal surgery in the elderly. World J Emerg Surg.

[23] Samuel JC, Qureshi JS, Mulima G, Shores CG, Cairns BA, Charles AG (2011). An Observational Study of the Etiology, clinical presentation and outcomes associated with peritonitis in Lilongwe, Malawi. World J Emerg Surg.

[24] Singh R, Kumar N, Bhattacharya A, Vajifdar H (2011). Preoperative predictors of mortality in adult patients with perforation peritonitis. Indian J Crit Care Med.

[25] Mawalla B, Mshana SE, Chalya PL, Imirzalioglu C, Mahalu W (2011). Predictors of surgical site infections among patients undergoing major surgery at Bugando Medical Centre in Northwestern Tanzania. BMC Surg.

[26] Kujath P, Schwandner O, Bruch HP (2002). Morbidity and mortality of perforated peptic gastroduodenal ulcer following emergency surgery. Langenbecks Arch Surg.

[27] Mäkelä JT, Kiviniemi H, Ohtonen P, Laitinen SO (2002). Factors that predict morbidity and mortality in patients with perforated peptic ulcers. European Journal of Surgery.

[28] Giiti GC, Mazigo HD, Heukelbach J, Mahalu W (2010). HIV, appendectomy and postoperative complications at a reference hospital in Northwest Tanzania: cross-sectional study. AIDS Res Ther.

